# Adult day services, leisure time satisfaction, and psychosocial well-being among dementia caregivers

**DOI:** 10.1093/geront/gnag163

**Published:** 2026-08-06

**Authors:** Clara J Scher, Jerad Moxley, Catherine Riffin, Sara Czaja

**Affiliations:** Division of Geriatrics and Palliative Medicine, Weill Cornell Medicine, New York, New York, United States; Division of Geriatrics and Palliative Medicine, Weill Cornell Medicine, New York, New York, United States; Division of Geriatrics and Palliative Medicine, Weill Cornell Medicine, New York, New York, United States; Division of Geriatrics and Palliative Medicine, Weill Cornell Medicine, New York, New York, United States

**Keywords:** Family caregivers, Community-based services, Mental health, Secondary data analysis

## Abstract

**Background and Objectives:**

Dementia caregivers experience high levels of emotional burden, and adult day services (ADS) may provide respite that allows for recovery from caregiving demands. Yet little is known about whether ADS respite contributes to greater leisure time satisfaction—an important aspect of caregivers’ respite experiences that may impact psychosocial well-being.

**Research Design and Methods:**

This secondary analysis used pooled longitudinal data from five national U.S. studies (REACH I, REACH II, Community REACH, Video to Video Network, and Caring for the Caregiving Network), totaling 2,417 dementia caregivers. Descriptive statistics and mixed-effects logistic and linear regression models with multiple imputation examined factors associated with ADS respite use and its longitudinal association with leisure time satisfaction, accounting for relevant covariates. Associations between leisure time satisfaction and psychosocial outcomes were examined.

**Results:**

ADS use was associated with more years of education and greater leisure time satisfaction among caregivers. Use of ADS respite was also associated with more functional limitations (IADLs) in the care recipient and less bother about care recipients’ IADL impairment among caregivers. Compared with non-use, ADS use was associated with higher leisure time satisfaction. Greater leisure time satisfaction was associated with less burden and loneliness as well as more social support and positive aspects of caregiving.

**Discussion and Implications:**

ADS use was associated with greater leisure time satisfaction, which in turn was related to caregiver well-being. Interventions that promote caregivers’ use of respite for satisfying activities and policies that expand access to ADS may maximize psychosocial benefits for dementia caregivers.

The number of people living with dementia in the United States is expected to nearly double by 2050, reaching approximately 13 million individuals (Alzheimer’s Association, 2025). Family caregivers are the primary source of support for this population. They provided an estimated 19.2 billion hours of care in 2024, translating to a financial value exceeding $413 billion (Alzheimer’s Association, 2025). As a result of caregiving demands (e.g., safety monitoring, bathing, dressing, medication management), dementia caregivers often face serious adverse outcomes, including caregiver burden (Brodaty et al., 2014), physical health conditions (Cho et al., 2024), social isolation and loneliness (Kovaleva et al., 2018), and limited opportunities for pleasurable activities (Ng et al., 2025). These stressors can lead to increased rates of anxiety (Kaddour & Kishita, 2020), depression (Collins & Kishita, 2020), and suicidality (Solimando et al., 2022).

Given the growing population of people living with dementia and their family caregivers (Alzheimer’s Association, 2025), there is an urgent need for effective respite services to support dementia caregivers’ health and well-being to sustain home-based dementia care (Utz, 2022). Respite—defined as time away from caregiving responsibilities (Lund et al., 2009)—is associated with less caregiver burden and distress among dementia caregivers (Coleman et al., 2025; Zarit et al., 2017). However, other studies have shown modest or no psychosocial benefits of respite (Neville et al., 2015; Vandepitte et al., 2016). Leisure time satisfaction—defined as caregivers’ perceived contentment with how leisure time is spent (Stevens et al., 2004)—may explain these inconsistencies, as dementia caregivers vary widely in how much they engage in activities that are personally satisfying during respite (Godfrey et al., 2025; Lund et al., 2009). One such source of caregiver respite is adult day services (ADS), which “support social, health, nutritional, and daily living needs of adults in congregate meal settings during day-time hours…enabl[ing] continued community-based living for individuals with physical and cognitive limitations and provid[ing] respite for their caregivers” (Anderson et al., 2013, p. 730). However, no research to date has used longitudinal, nationally representative data to evaluate whether dementia caregivers utilizing ADS for respite are satisfied with the quality of their leisure time.

Using a pooled dataset created by harmonizing outcomes across multiple longitudinal studies of dementia caregivers in the U.S., this study aimed to address this gap by examining the impact of ADS use on leisure time satisfaction among dementia caregivers. This study focuses on ADS given that psychosocial benefits (e.g., reduced burden and improved affect) of this service for caregivers are well documented (Zarit et al., 2017; Zarit, Kim, et al., 2014; Zarit, Whetzel, et al., 2014). Advancing understanding of caregivers’ satisfaction with leisure time is critical, as less contentment in respite activities may increase risk of negative psychosocial outcomes such as depression or anxiety (Vandepitte et al., 2016)—with serious implications not only for caregivers’ own well-being but also for the sustainability of a healthcare system which relies heavily on their unpaid support (Kuzuya et al., 2011).

## Health and social impact of dementia caregiving

Family caregivers of individuals with dementia experience stress, role overload, depression and anxiety, often at levels higher than non-caregivers and caregivers for other conditions (Cooper et al., 2007; Hellis & Mukaetova-Ladinska, 2022). Increased behavioral and psychological symptoms in the care recipient living with dementia can lead to more caregiver burden, depression, and anxiety (Sheehan et al., 2021), which often intensifies as the disease progresses (Mushtaq et al., 2025). In addition to mental health and psychosocial stressors, dementia caregiving is associated with increased prevalence of physical health conditions (Cho et al., 2024), including chronic pain (Turner et al., 2024), hypertension (Christian et al., 2023), cardiovascular disease (Xu et al., 2020), and diabetes (Laks et al., 2016).

There is also a significant social impact of dementia caregiving. Research has indicated that dementia caregiving can result in shrinking social networks and increasing isolation (Lee et al., 2022). Dementia caregivers report significant loneliness, especially as dementia severity advances, care recipients become homebound, or caregivers fear dementia-related stigma from others (Robertson et al., 2025). One population-based study in New Zealand suggested that objective isolation and subjective loneliness were both linked to poorer well-being in dementia caregivers (Robertson et al., 2025). On the other hand, social resources (e.g., social support from family and friends, relationships with service providers) can buffer the negative impacts of dementia caregiving on well-being (Xu et al., 2021).

### Dementia caregivers’ experiences of respite

Respite can be accessed through formal (e.g., adult day services, in-home, residential) or informal means (family and friends) (Godfrey et al., 2025; Neville et al., 2015). Several respite-related benefits can buffer against the negative impacts of dementia caregiving. For example, strong social support—which may be more accessible due to respite—is associated with less burden, fewer depressive and anxiety symptoms, better self-efficacy, and improved quality of life (Nasreen et al., 2024). In addition, achieving goals for leisure time use (e.g., engaging in activities prioritized by the caregiver) has been shown to increase dementia caregivers’ positive perceptions about their caregiving abilities (Godfrey et al., 2025). Yet, several challenges specific to the caregiver/care recipient (e.g., dyadic relations, guilt, anxiety, etc.) and the respite service (e.g., care quality, staff expertise, communication with caregivers, etc.) impact how dementia caregivers confer benefits from respite (O’Shea et al., 2019).

A large body of work has explored dementia caregivers’ experiences of respite time more generally, inclusive of respite obtained from both formal and informal service options. One study using nationally representative data in the U.S. found that dementia caregivers who used respite time to pursue activities at organizations (e.g., religious and spiritual practices) rated these activities as most pleasant, while productive activities (e.g., chores) were rated least pleasant (Ng et al., 2025). Engagement in pleasurable activities during respite time has been shown to be associated with positive impacts on mental health among dementia caregivers (Lee et al., 2020). For example, one study of 346 informal Australian dementia caregivers (88.15% female, age 18–82 years) found that leisure activities (e.g., being a member of a club) buffered the negative effects of caregiving (Schüz et al., 2015). Similarly, another study of 108 dementia caregivers in California who reported high levels of pleasant events (family time, going on outings) and low activity restriction had better psychosocial health outcomes (e.g., less depressive symptoms and role overload) compared to those who did not (Mausbach et al., 2011).

### Impact of adult day services (ADS) on the well-being dementia caregivers

Although research on the demographic characteristics of dementia caregivers using ADS respite remains limited, existing studies provide some insight into the populations most likely to use these services. In a 34-site ADS trial (ADS Plus), dementia caregivers were predominantly well-educated women (Gitlin et al., 2024). In addition, several care recipient and contextual factors are shown to influence ADS respite use among dementia caregivers, including caring for care recipients with greater functional limitations (e.g., ADL/IADL disability) and experiencing higher caregiver burden (Lee et al., 2022).

In addition, research has explored the effect of ADS respite on psychosocial well-being among dementia caregivers, demonstrating consistent benefits including reduced care-related stressors, improved mood, and lowered anger (Zarit, Kim, et al., 2014). One such study using a quasi-experimental design found that caregivers who used ADS respite had reduced overload, strain, depression, and anger (Zarit et al., 1998). Similarly, daily diary work with ADS users demonstrates that more objective respite (respite hours) related to increased positive affect, whereas more subjective respite (e.g., perceived breaks from caregiving) was associated with reduced negative affect (Wylie et al., 2021). In addition, related work has also shown that dementia caregivers’ use of ADS respite results in physiological improvements that support a more adaptive stress response to caregiving demands (Zarit, Whetzel, et al., 2014) and better sleep quality (Liu et al., 2022). These associations between ADS respite and reductions in stress have been hypothesized to be due, in part, to reduced exposure to care recipients’ psychological and behavioral symptoms of dementia (Gaugler et al., 2003).

Given that ADS serves a racially and ethnically diverse population of people living with dementia and their caregivers (Brown et al., 2014; Sadarangani et al., 2024), some research has focused on outcomes within historically minoritized groups. For example, Parker et al. (2019), Parker & Gitlin (2021) found that use of ADS respite was associated with lower depressive symptoms (Parker & Gitlin, 2021) and less likelihood to miss a physician appointment among dementia caregivers racialized as Black (Parker et al., 2019). In addition, ADS Plus, a multicomponent intervention embedded in ADS settings serving racially and ethnically diverse dementia caregivers, has also resulted in minimized depressive symptoms among dementia caregivers compared to usual care (Gitlin et al., 2024).

Yet, some older studies indicate no or very small between-group differences in effects of ADS on dementia caregiver psychosocial outcomes (e.g., depressive symptoms, quality of life) (Mossello et al., 2008; Warren et al., 2003; Zarit, Kim, et al., 2014). There could be several reasons for this. For example, dementia caregivers may respond differently to ADS respite depending on a variety of individual (e.g., guilt, burden) or organizational-level factors (e.g., trust in ADS staff). There could also be variation in the extent to which caregivers are engaging in satisfying activities during respite afforded through ADS, which may in turn have an effect on psychosocial well-being (Godfrey et al., 2025; Tretteteig et al., 2017).

### Impact of ADS on leisure time satisfaction among dementia caregivers

There is limited research focusing on leisure time satisfaction specific to dementia caregivers whose utilize ADS respite. Prior work has anecdotally described dementia caregivers using time afforded by ADS for rest, health appointments, work, practical tasks, socializing, and self-care (Tretteteig et al., 2016). Only two small, qualitative descriptive studies in Norway (Tretteteig et al., 2017) and Canada (Fortune & Gauthier-Gilbert, 2025) have explored the perspectives of dementia caregivers regarding the extent to which ADS meet their needs for support and respite. Findings suggested that using ADS respite helped caregivers feel more social support as well as enhance their ability to engage in activities such as physical activity, getting out of the home, spending time with family and friends, getting work done, and resting. In addition, one study conducted in 2010 with caregivers (not specific to dementia) in three ADS in the U.S. found that 46% of caregivers were only somewhat or not very satisfied with how they had spent their respite time after tracking for one week (Lund et al., 2009). As such, longitudinal, quantitative work to advance understanding of the causal and temporal associations between ADS respite and leisure time satisfaction is warranted.

### Conceptual framework

Guided by the Stress Process Model for Caregivers (Pearlin et al., 1990) and an ecological framework of recovery and leisure (Iwasaki et al., 2010), we conceptualize leisure time as a critical recovery experience from the behavioral and psychological symptoms of dementia and a source of resilience (e.g., increasing social engagement) for dementia caregivers. The Stress Process Model emphasizes how caregiver well-being is shaped by the interplay of contextual resources (e.g., socioeconomic status, family networks, and service availability), primary stressors (e.g., care recipient’s functional limitations, caregiver role overload), and secondary strains that arise both interpersonally (e.g., family conflict) and intrapsychically (e.g., diminished self-esteem). These stressors negatively influence caregiver mental and physical health outcomes, with coping strategies and social support serving as central mediators in this pathway. Complementing this perspective, the Recovery and Leisure Framework positions respite as an ecological recovery process, where engaging in enjoyable and meaningful activities fosters positive affect, coping, social connection, and physical health, ultimately supporting recovery, active living, and quality of life in the context of macro-level factors (e.g., cultural, environmental, and health-care system).

Taken together, these frameworks suggest that ADS may serve as a structural coping resource that improves caregiver psychosocial outcomes by providing respite through safe, supervised care for people living with dementia. By freeing caregivers’ time, ADS participation creates opportunities to engage in desired activities, thereby increasing the quality of and satisfaction with leisure time. Greater satisfaction with leisure time could enhance psychosocial well-being (e.g., depressive symptoms, positive aspects of caregiving, etc.). Thus, ADS participation may not only reduce direct stressors but also strengthen resilience through improved satisfaction with leisure time, ultimately promoting better outcomes.

### The current study

While prior research demonstrates psychosocial benefits of ADS for dementia caregivers, less attention has been paid to the characteristics of caregivers who utilize ADS respite, the association between ADS participation and satisfaction with leisure time, and the relationship between leisure time satisfaction and psychosocial well-being. To date, dementia caregivers’ leisure time satisfaction has not been systematically measured using longitudinal, quantitative methods among dementia caregivers using ADS respite (Tretteteig et al., 2017; Utz, 2022). To address this gap, this study sought to address three primary research questions:

1. *RQ1: What sociodemographic and contextual factors are associated with ADS respite use?* We hypothesize that several factors will be associated with ADS respite use among dementia caregivers, including being female, having a care recipient with greater functional limitations, experiencing higher caregiver burden, and having higher levels of education.
2. *RQ2: Compared to non-use, is use of ADS respite linked to leisure time satisfaction over time?* We hypothesize that ADS use will be associated with increased leisure time satisfaction compared to non-use among dementia caregivers.
3. *RQ3: Is there a psychosocial benefit of leisure time satisfaction for dementia caregivers over time?* We hypothesize that increased leisure time satisfaction will be associated with fewer depressive symptoms, loneliness, and burden, as well as more social support and positive aspects of caregiving.

Findings can inform if, and for whom, ADS participation facilitates leisure time satisfaction as well as future intervention research related to optimizing leisure time satisfaction among dementia caregivers using ADS respite.

## Method

### Data

This study is a secondary analysis of a longitudinal dataset that pooled five national studies of dementia caregivers, including REACH (Belle et al., 2003; Gitlin et al., 2003), REACH II (Belle et al., 2006), Community REACH (Czaja et al., 2018), Video to Video Network (Czaja et al., 2013), and Caring for the Caregiver Network (Lin et al., 2023) (See Supplementary Table 1 for a summary of characteristics for each study). REACH I used a multi-arm randomized clinical trial design to test a variety of caregiver interventions, demonstrating the feasibility of multi-component caregiver interventions with a sample of approximately 1,200 caregiver-care recipient dyads. Building on the findings of REACH I, REACH II was a randomized controlled trial testing a multi-component psychosocial intervention versus usual care among about 600 racially and ethnically diverse dementia caregivers. Community REACH involved a translation of REACH II delivered by community providers to about 146 ethnoracially diverse dementia caregivers, demonstrating the feasibility and effectiveness of translating REACH II into community settings. The Video-to-Video Network Study focused on 110 primarily Hispanic and African American dementia caregivers and involved a technology-delivered intervention modeled after REACH II over about 5 months. Finally, the Caring for the Caregiver Network Study was a randomized trial of technology-based psychosocial intervention among 243 dementia caregivers, demonstrating associations between the intervention and improved self-care, social support, and burden and depression.

These datasets were combined for the current study to create one longitudinal dataset of dementia caregivers in the U.S. collected from 1995 (REACH I) to 2018 (Caring for the Caregiver Network). This dataset was constructed from the five national datasets designed within the same research network that intentionally employed standardized measurement protocols (e.g., identical items, response options, and scoring procedures). Across studies, data were collected at multiple timepoints (e.g., baseline, post-intervention, and follow-ups), resulting in an unbalanced longitudinal dataset with repeated observations per caregiver (range = 1–4 timepoints, mean = 2.9 timepoints). Time was modeled as a linear variable and coded as months since baseline, enabling harmonization of measurement occasions and consistent alignment of observations across studies despite differences in assessment timing.

### Primary measures

#### Attending adult day services (ADS)

We determined whether or not caregivers utilized ADS using the Use of Formal Care and Services Questionnaire (Wisniewski et al., 2003). Caregivers were asked, “In the past month, did you or your care recipient attend an adult day care or adult day health program?” (Yes, No, or Unknown). If respondents answered yes, they were asked to specify which one of them received the service (Caregiver, Care Recipient or Both). Caregiver attendance responses were excluded because ADS are a care recipient-focused service that provides respite to caregivers; therefore this category was rarely endorsed in the sample. As a result, caregiver attendance was excluded to ensure a clear comparison of exposure groups (e.g., ADS use vs non-ADS use).

#### Leisure time satisfaction

The primary outcome was leisure time satisfaction, measured by the Leisure Time Satisfaction Scale (LTSS), which provides a standardized approach to assessing the quality and satisfaction of caregivers’ leisure experiences (Stevens et al., 2004). On a scale of not at all, a little, or a lot, respondents were asked how often they were able to spend time doing the following leisure activities: having quiet time by yourself, visiting family and friends, going out for meals, doing fun things with people, taking part in hobbies, sports, and attending church and other group activities. Each item ranged from 0 (none at all) to 2 (a lot), yielding a range of 0–14 for the total score, with higher scores representing more satisfaction with leisure (respite) time experiences.

### Covariates and secondary outcomes

Covariates related to the caregiver included age, years of education, gender (male or female), married (yes/no), widowed (yes/no), race/ethnicity (White, Black, Latinx, or other), and relationship to care recipient (spouse, child, or other). Mental health and support variables included loneliness, depressive symptoms, and social support. Loneliness was measured by a single item from the CESD-10 depressive symptoms scale (e.g., I felt lonely; rated as either rarely or none of the time, some or a little of the time, occasionally or a moderate amount of time, or all of the time) (Radloff, 1977). To examine loneliness as a distinct psychosocial outcome, the loneliness item was removed from the CES-D, while the remaining CES-D items were retained and summed to calculate depressive symptoms. Loneliness scores ranged from 0–3, with higher scores representing more days of experiencing loneliness per week. Depressive symptoms were scored from the other 9 items on the CES-D and ranged from 0–27, with higher scores representing more days expressing symptoms associated with depression. The social support questionnaire included three subscales measuring received support (e.g., number of relatives, friends or neighbors you can call on for help or feel close to), satisfaction with support (e.g., comfort, interest and concern from others), and non-supportive interactions (e.g., pried into affairs, taken advantage of you, been critical of you). The scale included 16 items, and the summed score ranged from 0–53, with higher scores representing greater social support and the negative interaction scale being reverse-coded.

Experiences of caregiving variables included years of caregiving, positive aspect of caregiving, caregiver burden (Bédard et al., 2001), and the extent to which the caregiver is bothered by the care recipient’s ADL (Katz et al., 1963) and IADL disability (Lawton & Brody, 1969). Years of caregiving were measured with the total number of years. Positive aspects of caregiving were a sum score from 11 items, with scores ranging from 0–44, with higher scores representing more agreement with positive statements about caregiving. Caregiver burden was the sum of 12 items and ranged from 0–48, with higher scores representing more frequent feelings of being burdened. Bothered by the care recipient’s ADLs was the sum of their rating of how much helping with each ADL bothered the caregiver, ranging from 0–24 with higher scores representing being extremely bothered by helping with each activity. Bothered by the care recipient’s IADLs was similarly coded for 8 items ranging 0–32.

Care recipient characteristics included age, gender, race/ethnicity, and ADL (Katz et al., 1963) and IADL disability (Lawton & Brody, 1969). The ADL score was a sum of 6 yes or no items and ranged from 0–6, with higher scores representing more activities the person needed help with. IADLs were similarly coded for 8 items ranging from 0–8. Finally, models also controlled for study type (Reach I, Reach II, Video to Video Network, Community Reach, and Caring for the Caregiver Network Study) and study arm (intervention vs control).

### Analytic strategy

Descriptive statistics, including means, standard deviations, and frequencies, were calculated using SPSS to summarize sample characteristics. Second, to understand which factors were associated with utilizing ADS respite, we fit binomial mixed-effects logistic regression models with a random intercept (e.g., accounting for the clustered structure of the data) in *R*. Missing data were addressed using multiple imputation in SPSS, generating five imputed datasets, and the regression analyses were conducted separately on each dataset. For each category of analysis, five imputed datasets were analyzed separately, and the resulting parameter estimates were combined using Rubin’s rules to produce pooled standard errors and statistical inferences (Rubin, 2009).

Next, we explored the longitudinal association between utilizing ADS and leisure time satisfaction by comparing dementia caregivers who utilized ADS with those who did not. A linear mixed model was estimated using SPSS, accounting for repeated measures and individual variability in change over time. To account for potential confounding related to study membership and study arm, study type and intervention assignment (intervention vs control) were included as covariates in all models. The outcome variable of leisure time satisfaction had a normal distribution. The model included random intercepts and slopes for time and controlled for participant and caregiver characteristics. Utilization of ADS respite (yes/no) was entered as a fixed predictor to examine its association with leisure time satisfaction, adjusting for the full set of covariates described above. Models were estimated using restricted maximum likelihood (REML) with Satterthwaite’s approximation for degrees of freedom.

Then, analyses using Python explored whether leisure time satisfaction was associated with several psychosocial outcomes (e.g., burden, depressive symptoms, social support, loneliness, positive aspects of caregiving), controlling for the full set of covariates described above. We used longitudinal linear mixed-effects regression models with multiple imputation, where leisure time satisfaction was a predictor, and each psychosocial variable was modeled as an outcome over time. Coefficients represented the average association between leisure time satisfaction and each outcome, accounting for repeated measures and covariates. Because results were pooled across multiple imputed mixed-effects models, statistical significance was again determined using Rubin’s rule to adjust the standard error and to shrink the degrees of freedom to account for variance associated with imputation. Because the primary objective of the present analyses was to examine associations between leisure time satisfaction and psychosocial outcomes, intervention assignment was included as a control variable rather than evaluated as a predictor.

To account for differences in assessment timing across studies, time was treated as a continuous variable and modeled using linear mixed-effects models, which allow for unequal numbers of observations and unequally spaced time points across participants. Random intercepts and slopes were included to capture individual variation in baseline levels of leisure time satisfaction and change over time. ADS utilization and leisure time satisfaction were modeled as time-varying measures assessed concurrently at each available wave. Psychosocial outcomes (e.g., loneliness, depressive symptoms) were also treated as time-varying, whereas all other variables (e.g., caregiver and care recipient characteristics, study characteristics) were included as baseline covariates.

## Results

Descriptive statistics are presented in Table 1. There were 2,417 caregivers of people living with dementia in the sample. Among those, the average leisure time satisfaction rating was 6.17 (range 0–14). About 26% of caregivers in the sample attended adult day (*n* = 306). The mean age of dementia caregivers was 61.16 (*SD* = 13.49; range 20–95), and the average years of education was 12.72 (*SD* = 2.95). Most caregivers were female (82%), about half were married (50%), and more than a third were widowed (39%). In terms of racial identity, about 42% identified as White, 30% identified as Latina/o, 27% identified as Black, and 1% as other. With respect to the relationship to the care recipient, approximately 45% were spouse caregivers while 44% were child caregivers. Caregivers rated their loneliness at an average of 0.95 (*SD* = 1.10) on a scale of 0 = rarely/none of the time to 3 = most/all of the time and depressive symptoms as 10.02 (*SD* = 4.44) on a scale of 0 to 26.1. Caregivers, on average, reported having moderate levels of social support (29.5; *SD* = 8.04; range 2.14 to 53). Regarding study participation, about 59% of caregivers were in the intervention group, 40% in the control group, and 1% were missing. Approximately 51% of participants came from Reach I, 28% from Reach II, 5% from Video-to-Video Network, 6% from Community Reach, and 10% from Caring for the Caregiver Network. On average, care recipients were 78.76 years old (*SD* = 8.71, range 42 to 103) and most were female (57%). In terms of racial identity, about 27% of care recipients identified as Black, 42% as White, 30% as Latinx, and 2% as other. The average number of ADLs was 3.36 (*SD* = 2.06; range 0 to 6), and the average number of IADLs was 6.72 (*SD* = 1.57; range 0 to 8).

**Table 1:** Descriptive statistics of the study sample.

Variables (*N* = 2,417)	Mean/% (Valid *N*)	*SD*	Min	Max
**Primary independent variable and outcome**				
Leisure time satisfaction	6.17 (2243)	3.53	0	14
Attended ADS (yes)	26.4 (306)			
**Caregiver demographics**				
Age	61.16 (2317)	13.49	20	95
Years of education	12.72 (2389)	2.95	0	17
Gender (female)	81.7 (1971)			
Married (yes)	49.7 (1188)			
Widowed (yes)	38.6 (922)			
**Race/ethnicity**				
Black	26.8 (641)			
White	42 (1002)			
Latinx	30.4 (726)			
Other	0.8 (20)			
Relationship to care recipient: spouse (yes)	44.8 (1080)			
Relationship to care recipient: child (yes)	44.3 (1068)			
**Caregiver mental health and support**				
Loneliness	0.95 (2389)	1.10	0	3
Depressive symptoms	10.02 (2390)	4.44	0	26.1
Social support	29.5 (2390)	8.04	2.14	53
**Experience of caregiving**				
Years of caregiving	4.62 (2383)	5.49	0	58
Positive aspects of caregiving	31 (2390)	10.75	0	44
Caregiver burden	19.03 (1161)	9.85	0	47
ADL bother	2.71 (1850)	4.04	0	24
IADL bother	3.95 (1799)	4.99	0	32
**Care recipient demographics**				
Age	78.76 (2314)	8.71	42	103
Gender (female)	57.4 (1331)			
** Race/ethnicity**				
Black	26.9 (651)			
White	41.8 (1011)			
Latinx	29.9 (713)			
Other	1.7 (42)			
ADLs	3.36 (2390)	2.06	0	6
IADLs	6.73 (2390)	1.57	0	8
**Study arm**				
Intervention	59 (1417)			
Control	40 (972)			
Missing	1 (28)			
**Studies**				
Reach I (yes)	50.8 (1229)			
Reach II (yes)	28 (670)			
Video to video network (yes)	5.3 (128)			
Community reach (yes)	6 (146)			
Caring for the caregiver network (yes)	10.1 (244)			

*Note*. ADS = adult day services, ADL = activities of daily living, IADL = instrumental activities of daily living.

To address our first research question, factors associated with ADS respite use are presented in Table 2. Higher educational attainment (*B *= 0.14, *SE* = 0.06, *p* = .05) and greater leisure time satisfaction (*B *= 0.19, *SE* = 0.05, *p* < .001) were associated with more ADS respite use among dementia caregivers. Greater care recipient functional impairment, as indicated by higher IADL limitations, was associated with greater ADS respite use (*B *= 0.24, *SE* = 0.05, *p* < .001). Being widowed (*B *= −0.29, *SE* = 0.14, *p* = .04) and higher levels of bother with IADL limitations (*B *= −0.12, *SE* = 0.05, *p* = .04) were associated with less ADS respite use. Time was significantly associated with ADS respite use (*B *= −0.14, *SE* = 0.06, *p* = .03), such that ADS use declined over the study period.

**Table 2:** Factors associated with utilization of ADS respite.

Variables	Estimate	*SE*	*t*-value	df	*p*(>|*t*|)
**Leisure time satisfaction**	0.19	0.05	3.48	15.41	.00***
**Caregiver demographics**					
Age	0.07	0.09	0.75	12.11	.47
Years of education	0.14	0.06	2.19	12.95	.05**
Gender (female) (ref = male)	0.17	0.16	1.05	19.60	.31
Married (yes) (ref = no)	−0.46	0.31	−1.47	9.52	.17
Widowed (yes) (ref = no)	−0.29	0.14	−2.10	198.36	.04**
**Race (ref = White)**					
Black	1.40	1.08	1.29	8.46	.23
Latinx	−0.19	0.42	−0.45	22.25	.66
Other	0.33	0.66	0.49	27.18	.63
Relationship to care recipient: spouse (yes) (ref = no)	0.30	0.18	1.68	10.95	.12
Relationship to care recipient: child (yes) (ref = no)	0.12	0.08	1.45	34.00	.16
**Caregiver mental health and support**					
Loneliness	−0.06	0.05	−1.31	78.45	.20
Depressive symptoms	−0.08	0.08	−1.02	8.25	.34
Social support	−0.06	0.06	−1.06	14.89	.30
**Experience of caregiving**					
Years of caregiving	−0.03	0.04	−0.72	63.03	.47
Positive aspects of caregiving	−0.05	0.06	−0.86	10.29	.41
Caregiver burden	0.27	0.11	2.44	6.00	.05
ADL bother	0.18	0.09	1.97	6.67	.09
IADL bother	−0.12	0.05	−2.18	27.10	.04**
**Care recipient demographics**					
Age	−0.02	0.07	−0.31	12.32	.76
Gender (female) (ref = male)	0.17	0.12	1.38	787.78	.17
** Race (ref = White)**					
Black	−0.97	1.10	−0.88	8.26	.40
Latinx	0.63	0.44	1.43	18.99	.17
Other	0.94	0.57	1.65	12.57	.12
ADLs	0.05	0.08	0.58	7.25	.58
IADLs	0.24	0.05	4.48	28.86	.00***
**Study arm (ref = control)**					
Intervention	0.19	0.10	1.89	26.73	.07
**Studies (ref = Reach 2)**					
Reach I (yes)	−0.16	0.14	−1.14	26.18	.26
Video to video network (yes)	−0.55	0.21	−2.64	1436.73	.01**
Community reach (yes)	−1.35	0.23	−5.80	669.25	.00***
Caring for the caregiver network (yes)	0.13	0.15	0.87	362.44	.39
**Timepoint**	−0.14	0.06	−2.21	41.35	.03**
**Intercept**	−1.30	0.24	−5.49	240.59	.00***
**Time × intervention**	−0.05	0.07	−0.62	49.86	.54

*Note*. ADS = adult day services, ADL = activities of daily living, IADL = instrumental activities of daily living.

*** *p* < .001.

** *p* < .05.

To address our second research question, we examined whether leisure time satisfaction differed over time between dementia caregivers who utilized ADS and those who did not. The results are summarized in Table 3. Compared to non-use, using ADS respite was significantly associated with greater leisure time satisfaction (*B *= 0.11, *SE* = 0.03, *p* < .001), controlling for the full set of covariates. Higher caregiver education (*B *= 0.07, *SE* = 0.02, *p* < .001), social support (*B *= 0.25, *SE* = 0.01, *p* < .001), and positive aspects of caregiving (*B *= 0.04, *SE* = 0.02, *p* = .01) were associated with higher leisure time satisfaction. Time was significantly associated with leisure time satisfaction (*B *= 0.13, *SE* = 0.02, *p* < .001), such that leisure time satisfaction increased over the study period. Higher loneliness (*B *= −0.05, *SE* = 0.01, *p* < .001) and caregiver burden (*B *= −0.23, *SE* = 0.03, *p* < .001) were associated with lower leisure time satisfaction. Higher ADLs (*B *= −0.13, *SE* = 0.01, *p* < .001) and IADLs (*B *= −0.04, *SE* = 0.01, *p* < .001) in the care recipient were associated with lower leisure time satisfaction for the caregiver.

**Table 3:** Association between utilizing ADS respite and leisure time satisfaction among dementia caregivers.

Variables	Estimate	*SE*	*t*	df	*p*(>|*t*|)
**Attended ADS (yes) (ref = no)**	0.11	0.03	3.59	17.41	.00***
**Caregiver demographics**					
Age	−0.02	0.02	−0.74	353.48	.46
Years of education	0.07	0.02	4.56	465.64	.00***
Gender (female) (ref = male)	−0.03	0.05	−0.65	431.31	.51
Married (yes) (ref = no)	0.04	0.07	0.57	206.90	.57
Widowed (yes) (ref = no)	0.04	0.05	0.94	44378.28	.35
**Race (ref = White)**					
Black	−0.58	0.22	−2.58	151.77	.01**
Latinx	0.06	0.12	0.49	651.38	.62
Other	−0.48	0.21	−2.32	234.59	.02**
Relationship to care recipient: spouse (yes) (ref = no)	−0.13	0.04	−3.03	111.11	.00***
Relationship to care recipient: child (yes) (ref = no)	−0.06	0.02	−2.30	647.90	.02**
**Caregiver mental health and support**					
Loneliness	−0.05	0.01	−3.71	140.13	.00***
Depressive symptoms	−0.03	0.02	−1.65	60.80	.10
Social support	0.25	0.01	18.43	108.22	.00***
**Experience of caregiving**					
Years of caregiving	0.00	0.01	−0.07	117.64	.94
Positive aspects of caregiving	0.04	0.02	2.74	30.81	.01**
Caregiver burden	−0.23	0.03	−8.73	7.71	.00***
ADL bother	0.02	0.02	0.97	52.39	.34
IADL bother	0.01	0.02	0.90	49.39	.37
**Care recipient demographics**					
Age	−0.04	0.02	−1.80	33.80	.08
Gender (female) (ref = male)	−0.09	0.04	−2.02	593.79	.04**
** Race (ref = White)**					
Black	0.35	0.22	1.62	246.76	.11
Latinx	−0.16	0.12	−1.32	346.19	.19
Other	0.09	0.16	0.57	200.22	.57
ADLs	−0.13	0.01	−8.75	95.98	.00***
IADLs	−0.04	0.01	−3.49	771.08	.00***
**Study arm (ref = control)**					
Intervention	0.03	0.03	1.18	2783.79	.24
**Studies (ref = Reach 2)**					
Reach I (yes)	0.16	0.04	3.94	196.33	.00***
Video to video network (yes)	−0.15	0.07	−2.14	5503.31	.03**
Community reach (yes)	0.01	0.08	0.11	39.05	.91
Caring for the caregiver network (yes)	0.35	0.05	6.54	2507.74	.00***
**Timepoint**	0.13	0.02	6.54	22.12	.00***
**Intercept**	−0.06	0.08	−0.67	1201.33	.50
**Time × intervention**	0.00	0.02	0.19	195.33	.85

*Note.* ADS = adult day services, ADL = activities of daily living, IADL = instrumental activities of daily living.

*** *p* < .001.

** *p* < .05.

To address our third research question, associations between leisure time satisfaction and psychosocial outcomes are presented in Supplementary Table 2. Across a series of separate multivariable mixed-effects models (full models available upon request), greater leisure time satisfaction was consistently associated with less burden (*B *= −0.16, *SE* = 0.02, *p* < .001), less loneliness (*B *= −0.04, *SE* = 0.01, *p* < .001), greater social support (*B *= 0.21, *SE* = 0.01, *p* < .001), and higher positive aspects of caregiving (*B *= 0.04, *SE* = 0.01, *p* < .01). Leisure time satisfaction was not associated with depressive symptoms.

## Discussion

This paper advances knowledge on the importance of dementia caregivers’ satisfaction with time spent during respite (e.g., leisure time) afforded by adult day services (ADS). Findings suggested that dementia caregivers who utilized ADS respite had greater leisure time satisfaction over time compared to those who did not. In separate analyses, leisure time satisfaction was associated with psychosocial benefits (e.g., less burden, lower loneliness, greater positive aspects of caregiving). These results are aligned with the Stress Process Model of Caregiving (Pearlin et al., 1990) and ecological framework of recovery and leisure (Iwasaki et al., 2010) such that ADS respite may act as support that allows caregivers temporary relief from caregiving demands, facilitating burden reduction and restorative experiences that enhance coping and recovery (e.g., social support). This work addresses a gap by examining leisure time satisfaction among dementia caregivers who use ADS using a longitudinal and nationally representative sample of dementia caregivers in the U.S. Dementia caregivers provide substantial unpaid care for individuals living with dementia in the community and contribute significantly to the broader healthcare system (Hurd et al., 2015; Lastuka et al., 2025; Nandi et al., 2024). At the same time, dementia caregiving is also associated with increased mental and physical health conditions and subsequent healthcare utilization among caregivers themselves (Martindale-Adams et al., 2016; Zhu et al., 2015). Therefore, understanding and optimizing the satisfying nature of respite for this population may help support caregivers’ well-being and the sustainability of caregiving roles over time (Utz, 2022; Zarit et al., 2017).

There were three key findings of this study. First, we found that several characteristics of dementia caregivers were associated with more ADS respite utilization, including those who were adult child caregivers and spousal caregivers, as well as those who were not widowed. Having higher levels of education and leisure time satisfaction were also associated with greater ADS respite use. In addition, having a care recipient with higher IADL limitations was associated with greater ADS respite use. Higher ADS use among caregivers whose care recipients have more IADL—but not ADL—impairment could reflect that ADS programs are often better suited to addressing moderate functional (e.g., medication management) rather than intensive personal care needs (e.g., toileting, continence). Our findings align with prior literature exploring caregiver characteristics associated with respite use. Though not specific to the ADS setting, multiple studies drawing on respite service use data from the nationally representative datasets on caregiving similarly found that caregivers whose care recipients had higher levels of functional limitations were more likely to use respite services (Lee et al., 2022; Ogunjesa et al., 2024; Riffin et al., 2019). In addition, multiple studies have demonstrated that adult children caregivers and female spouses who are not widowed are more likely to utilize ADS respite (Stirling et al., 2014; Tretteteig et al., 2016).

Second, controlling for a set of caregivers- and care recipient-related characteristics, we found that compared to non-users, utilizing ADS respite is associated with more leisure time satisfaction over time among dementia caregivers. One possible explanation for the higher leisure time satisfaction observed among ADS users is that ADS often provide longer, more structured and predictable respite hours compared to other forms of non-ADS respite (e.g., intermittent in-home assistance or informal support), therefore affording caregivers more meaningful and restorative time away from caregiving responsibilities (Wylie et al., 2021). Although this study is, to our knowledge, the first to examine this association using longitudinal data, a few prior qualitative descriptive studies and related quantitative studies provide supporting evidence. For example, qualitative studies and integrative reviews of dementia-focused adult day centers report that ADS respite allows caregivers to experience a feeling of freedom and more time for their own needs, social activities, and relaxation (Tretteteig et al., 2016, 2017). In addition, daily diary work with caregivers utilizing ADS respite shows that caregivers received substantially more respite time on ADS days (e.g., 7.1 vs 1.7 hours), and higher objective and subjective breaks from caregiving was associated with higher positive mood (Wylie et al., 2021).

In contrast, broader research not only specific to ADS highlights that some caregivers feel that they “waste” respite time and report low satisfaction with how it is used (Godfrey et al., 2025; Neville et al., 2015). Factors such as guilt, fatigue, and lack of trust in respite care providers act as barriers to using respite time for satisfying activities (Fortune & Gauthier-Gilbert, 2025). This could suggest that caregivers’ satisfaction with their respite time depends on how they choose to use respite time (Godfrey et al., 2025). While our findings did not necessarily reflect this, it could be that ADS is one accessible, trusted, community-based support service that is well positioned to promote engagement in satisfying activities during respite. Importantly, we also found that ADS use declined but leisure time satisfaction increased over the study period. This pattern may reflect changes in caregiving trajectories overtime, including care recipient death or transitions to higher levels of care (e.g., nursing homes), which could reduce ADS use and impact caregivers’ opportunities for and perceptions of their leisure time satisfaction.

Third, results indicated that leisure time satisfaction was independently associated with several psychosocial benefits for dementia caregivers, including less burden, lower loneliness, greater perceived social support, and higher positive aspects of caregiving. These findings are aligned with results of studies examining the Time for Living and Caring Intervention—an online coaching app designed to encourage caregivers to engage in activities that are desirable to them during respite (Coleman et al., 2025; Godfrey et al., 2025; Iacob et al., 2024)—which has been shown to increase positive aspects of caregiving. In addition, early work exploring perspectives of 52 family caregivers who used ADS similarly found that those who did not consistently complete the activities they desired during respite had lower satisfaction with respite time, were more negative in dimensions of burden, and less satisfied with caregiving (Lund et al., 2009). In addition, another study found that perceived restriction of leisure time mediated the association between caregiver burden and mental health (e.g., depressive symptoms, anxiety, lower life satisfaction) and that engaging in social groups (e.g., clubs, religious organizations, etc.) diminished the negative mental health effects of caregiving (Schüz et al., 2015).

Notably, in the current study, leisure time satisfaction was not longitudinally associated with depressive symptoms. This finding is in contrast to other studies—not specific to ADS respite (e.g., home-based respite)—finding that more frequent and higher satisfaction in respite time use was associated with lower depressive symptoms (Lee et al., 2020; Mensie & Steffen, 2010). However, our findings are aligned with prior reviews in the dementia caregiving literature that have similarly reported inconsistent effects of respite use on depressive symptoms, due to heterogeneous and weak evidence, variation in respite types and dosage, and different outcome measurement approaches (Neville et al., 2015). Alternatively, this null finding may reflect that leisure time satisfaction provides a short-term feeling of relief from caregiving demands more closely tied to positive mood than to the long-lasting nature of depressive symptoms.

### Limitations

Several limitations of the current study should be acknowledged. First, because mixed-effects models estimate average associations across time, the observed relationships cannot be interpreted as causal, and directionality may be bi-directional (e.g., lower caregiver burden may facilitate greater satisfaction with respite, rather than satisfaction with respite leading to lower caregiver burden). Second, we did not explicitly account for care recipients who died or transitioned to institutional care during the study period, nor were we able to formally examine differential attrition or changes in caregiving context over time. These factors may have influenced patterns of ADS use and leisure time satisfaction across follow-up assessments and should therefore be considered when interpreting the longitudinal results. Further, several potentially important confounders, including dementia subtype, behavioral symptoms, financial resources, and use of other formal or community-based supports, were not available in a harmonized form across all parent datasets and therefore could not be included in the models, which may result in residual confounding. Third, this study relied on secondary analysis of pooled data from five national studies that were not originally designed to examine ADS respite use in depth. Although the parent studies included items assessing the types of leisure activities caregivers engaged in during respite and the frequency with which they engaged in them, these indicators were combined into a continuous measure of leisure time satisfaction to capture caregivers’ overall appraisal of their respite experiences. As a result, the present analyses focused on a global satisfaction with leisure time rather than disentangling the specific activities or patterns of use that may differentially influence psychosocial outcomes—an objective that was beyond the scope of the current study.

In addition, the questionnaire item assessing ADS use did not distinguish between social and medical programs, which prevented us from examining whether outcomes differed by ADS model type. Another limitation of this study is the small proportion of Asian-identified caregivers in the sample and the inability to disaggregate the “other” racial category, which limited examination of heterogeneity within racial groups. Finally, despite adjustment for relevant covariates and use of multiple imputation, the harmonized dataset combined studies across different geographic regions and historical periods; therefore, unmeasured differences in ADS availability, service access, and broader caregiving contexts may have influenced both ADS use and caregiver outcomes. Therefore, residual heterogeneity may remain despite the use of linear and logistic mixed-effects models and adjustment for study characteristics and participant-level covariates.

### Implications for future research

The findings of this study indicate several implications for future research. For example, future work should use quantitative, longitudinal methods to explore characteristics associated with differential trajectories of leisure time satisfaction over time among dementia caregivers utilizing ADS respite (Fortune & Gauthier-Gilbert, 2025). For example, factors such as relationship type (e.g., spousal, child, or other non-kin caregivers) and relationship quality with care recipients may affect time spent on and enjoyment of non-caregiving activities (Ng et al., 2025). In addition, future work should explore the specific activities or patterns of leisure time use (e.g., participation in community groups vs time spent alone to rest) that may differentially influence psychosocial outcomes among dementia caregivers. Complementary qualitative methods can center the voices of dementia caregivers utilizing ADS to advance understanding of barriers to leisure time satisfaction such as guilt (Neville et al., 2015), cultural beliefs (Lee et al., 2022), and employment responsibilities, as well as facilitators such as social support and trust in ADS staff (Young et al., 2024). Second, research should explore ADS providers’ perspectives on what strategies could be used to enhance caregivers’ leisure time satisfaction as well as potential organizational barriers to implementing this in ADS settings (Anderson et al., 2015; Gitlin et al., 2025). While online interventions focused on respite time satisfaction exist (Godfrey et al., 2025), these approaches are not yet tailored to or embedded within ADS settings where providers can leverage their relationship with caregivers and resources at the center. In addition, though dementia caregiver interventions have been integrated into ADS settings, these interventions do not focus on leisure time use or satisfaction, and instead incorporate dementia education, care skills training, and community referrals (Gitlin et al., 2025). Future work can inform development of ADS-embedded strategies to enhance leisure time satisfaction that are both responsive to dementia caregivers’ unique needs and also feasible for ADS providers to implement given resource constraints and time pressures (Anderson et al., 2013; Sadarangani et al., 2022; Scher et al., 2025).

### Implications for practice

Results of the current study highlight the potential importance of leisure time satisfaction as a potential mechanism for improving psychosocial outcomes among dementia caregivers who are ADS respite users. Therefore, ADS providers (e.g., administrative or clinical staff) could consider having regular, informal conversations with dementia caregivers about how they are using their leisure time, including prompting caregivers to assess their respite needs, setting goals around specific activities they want to complete, and engaging in goal review (Iacob et al., 2024; Utz et al., 2023). However, more research is needed to understand the organizational factors that enable or constrain ADS providers from incorporating interventions to enhance leisure time satisfaction for dementia caregivers into routine practice.

## Conclusion

Results of the current study emphasize the role of leisure time satisfaction—made possible by protected respite time through ADS—in promoting dementia caregivers’ psychosocial well-being. Given the tremendous healthcare costs associated with caring for people living with dementia (Hurd et al., 2015), and the integral role of dementia caregivers in sustaining our healthcare system (Kuzuya et al., 2011), our findings strengthen the case for policy supports for ADS—an industry that serves as a structural coping resource for dementia caregivers. Advocacy organizations such as the National Adult Day Services Association and LeadingAge position ADS as a vital part of the continuum of home- and community-based long-term care, arguing for expanding accessibility and access to ADS across federal funding streams such as Medicaid, Medicare, Older Americans Act Programs, and Veterans Affairs (LeadingAge, n.d.; NADSA, n.d.). These efforts are critical to ensuring that ADS remain accessible and adequately funded for dementia caregivers to support their well-being and allow care recipients to remain aging in community.

## Supplementary material

Supplementary material is available at *The Gerontologist* online.

## Supplementary Material

gnag163_Supplementary_Data

## Data Availability

The data from this study are available from the authors upon reasonable request. This study was not preregistered.

## References

[gnag163-B1] Alzheimer’s Association. (2025). 2025 Alzheimer’s disease facts and figures. Alzheimer’s & Dementia, 21, e70235. 10.1002/alz.70235 22404854

[gnag163-B2] Anderson K. A. , Dabelko-Schoeny H. I., Fields N. L., Carter J. R. (2015). Beyond respite: The role of adult day services in supporting dementia caregivers. Home Health Care Services Quarterly, 34, 101–112. 10.1080/01621424.2015.1040939 25894489

[gnag163-B3] Anderson K. A. , Dabelko-Schoeny H., Johnson T. D. (2013). The state of adult day services: Findings and implications from the MetLife National Study of Adult Day Services. Journal of Applied Gerontology: The Official Journal of the Southern Gerontological Society, 32, 729–748. 10.1177/0733464812447284 25474796

[gnag163-B4] Bédard M. , Molloy D. W., Squire L., Dubois S., Lever J. A., O’Donnell M. (2001). The Zarit Burden Interview: A new short and screening version. The Gerontologist, 41, 652–657. 10.1093/geront/41.5.652 11574710

[gnag163-B5] Belle S. H. , Burgio L., Burns R., Coon D., Czaja S. J., Gallagher-Thompson D., Gitlin L. N., Klinger J., Koepke K. M., Lee C. C., Martindale-Adams J., Nichols L., Schulz R., Stahl S., Stevens A., Winter L., Zhang S. REACH II Investigators. (2006). Enhancing the quality of life of dementia caregivers from different ethnic or racial groups: A randomized controlled trial. Annals of Internal Medicine, 145, 727–738. 10.7326/0003-4819-145-10-200611210-00005 PMC2585490 17116917

[gnag163-B6] Belle S. H. , Czaja S. J., Schulz R., Zhang S., Burgio L. D., Gitlin L. N., Jones R., Mendelsohn A. B., Ory M. G. REACH Investigators. (2003). Using a new taxonomy to combine the uncombinable: Integrating results across diverse interventions. Psychology and Aging, 18, 396–405. 10.1037/0882-7974.18.3.396 PMC2579277 14518803

[gnag163-B7] Brodaty H. , Woodward M., Boundy K., Ames D., Balshaw R. PRIME Study Group. (2014). Prevalence and predictors of burden in caregivers of people with dementia. The American Journal of Geriatric Psychiatry: Official Journal of the American Association for Geriatric Psychiatry, 22, 756–765. 10.1016/j.jagp.2013.05.004 24012226

[gnag163-B8] Brown E. L. , Friedemann M.-L., Mauro A. C. (2014). Use of adult day care service centers in an ethnically diverse sample of older adults. Journal of Applied Gerontology: The Official Journal of the Southern Gerontological Society, 33, 189–206. 10.1177/0733464812460431 24652954

[gnag163-B9] Cho J. , Sands L. P., Stevens A. B., Allore H. G., Horstman M. J. (2024). Profile of caregiving activities and association with physical health among dementia spousal caregivers. Innovation in Aging, 8, igae017. 10.1093/geroni/igae017 PMC10960627 38524243

[gnag163-B10] Christian L. M. , Wilson S. J., Madison A. A., Prakash R. S., Burd C. E., Rosko A. E., Kiecolt-Glaser J. K. (2023). Understanding the health effects of caregiving stress: New directions in molecular aging. Ageing Research Reviews, 92, 102096. 10.1016/j.arr.2023.102096 PMC10824392 37898293

[gnag163-B11] Coleman M. E. , Godfrey D. A., Thompson A. D., Sparks C., Utz R. L. (2025). Improving respite outcomes for caregivers: Who benefits and under what conditions? Journal of Applied Gerontology: The Official Journal of the Southern Gerontological Society, 44, 1913–1920. 10.1177/07334648251324270 PMC12353123 40067065

[gnag163-B12] Collins R. N. , Kishita N. (2020). Prevalence of depression and burden among informal caregivers of people with dementia: A meta-analysis. Ageing and Society, 40, 2355–2392. 10.1017/S0144686X19000527

[gnag163-B13] Cooper C. , Balamurali T. B. S., Livingston G. (2007). A systematic review of the prevalence and covariates of anxiety in caregivers of people with dementia. International Psychogeriatrics, 19, 175–195. 10.1017/S1041610206004297 17005068

[gnag163-B14] Czaja S. J. , Lee C. C., Perdomo D., Loewenstein D., Bravo M., Moxley J. H., Schulz R. (2018). Community REACH: An implementation of an evidence-based caregiver program. The Gerontologist, 58, e130–e137. 10.1093/geront/gny001 29562361

[gnag163-B15] Czaja S. J. , Loewenstein D., Schulz R., Nair S. N., Perdomo D. (2013). A videophone psychosocial intervention for dementia caregivers. The American Journal of Geriatric Psychiatry: Official Journal of the American Association for Geriatric Psychiatry, 21, 1071–1081. 10.1016/j.jagp.2013.02.019 23831174

[gnag163-B16] Fortune D. , Gauthier-Gilbert A. (2025). Addressing caregivers’ needs beyond respite: Enhancing social connections and letting them know they matter. Canadian Journal on Aging = La Revue Canadienne du Vieillissement, 44, 403–412. 10.1017/S0714980825100160 40735824

[gnag163-B17] Gaugler J. E. , Jarrott S. E., Zarit S. H., Stephens M.-A. P., Townsend A., Greene R. (2003). Respite for dementia caregivers: The effects of adult day service use on caregiving hours and care demands. International Psychogeriatrics, 15, 37–58. 10.1017/S1041610203008743 12834199

[gnag163-B18] Gitlin L. N. , Burgio L. D., Mahoney D., Burns R., Zhang S., Schulz R., Belle S. H., Czaja S. J., Gallagher-Thompson D., Hauck W. W., Ory M. G. REACH Investigators. (2003). Effect of multicomponent interventions on caregiver burden and depression: The REACH multisite initiative at 6-month follow-up. Psychology and Aging, 18, 361–374. 10.1037/0882-7974.18.3.361 PMC2583061 14518800

[gnag163-B19] Gitlin L. N. , Marx K. B., Roth D. L., Anderson K., Dabelko-Schoeny H., Scerpella D., Parker L. J., Koeuth S., Gaugler J. E. (2025). Fidelity matters: Implementing ADS Plus, an evidence-based program, in multiple adult day service sites. Innovation in Aging, 9, igaf074. 10.1093/geroni/igaf074 PMC12448453 40979469

[gnag163-B20] Gitlin L. N. , Roth D. L., Marx K., Parker L. J., Koeuth S., Dabelko-Schoeny H., Anderson K., Gaugler J. E. (2024). Embedding caregiver support within adult day services: Outcomes of a multisite trial. The Gerontologist, 64, gnad107. 10.1093/geront/gnad107 PMC10943495 37549428

[gnag163-B21] Godfrey D. A. , Wong B., Thompson A. D., Coleman M. E., Sparks C., Utz R. L. (2025). Respite time-use among dementia caregivers. Frontiers in Health Services, 5, 1598518. 10.3389/frhs.2025.1598518 PMC12336254 40792175

[gnag163-B22] Hellis E. , Mukaetova-Ladinska E. B. (2022). Informal caregiving and Alzheimer’s disease: The psychological effect. Medicina (Kaunas, Lithuania), 59, 48. 10.3390/medicina59010048 PMC9863258 36676672

[gnag163-B23] Hurd M. D. , Martorell P., Langa K. (2015). Future monetary costs of dementia in the United States under alternative dementia prevalence scenarios. Journal of Population Ageing, 8, 101–112. 10.1007/s12062-015-9112-4 PMC4410878 25926904

[gnag163-B24] Iacob E. , Caserta M., Donaldson G., Sparks C., Terrill A., Thompson A., Wong B., Utz R. L. (2024). Evaluating the efficacy of Time for Living and Caring: An online intervention to support dementia caregivers’ use of respite. Innovation in Aging, 8, igae043. 10.1093/geroni/igae043 PMC11129597 38803611

[gnag163-B25] Iwasaki Y. , Coyle C. P., Shank J. W. (2010). Leisure as a context for active living, recovery, health and life quality for persons with mental illness in a global context. Health Promotion International, 25, 483–494. 10.1093/heapro/daq037 PMC2979346 20543204

[gnag163-B26] Kaddour L. , Kishita N. (2020). Anxiety in informal dementia carers: A meta-analysis of prevalence. Journal of Geriatric Psychiatry and Neurology, 33, 161–172. 10.1177/0891988719868313 31409196

[gnag163-B27] Katz S. , Ford A. B., Moskowitz R. W., Jackson B. A., Jaffe M. W. (1963). Studies of illness in the aged. The index of ADL: A standardized measure of biological and psychosocial function. JAMA, 185, 914–919. 10.1001/jama.1963.03060120024016 14044222

[gnag163-B28] Kovaleva M. , Spangler S., Clevenger C., Hepburn K. (2018). Chronic stress, social isolation, and perceived loneliness in dementia caregivers. Journal of Psychosocial Nursing and Mental Health Services, 56, 36–43. 10.3928/02793695-20180329-04 29667698

[gnag163-B29] Kuzuya M. , Enoki H., Hasegawa J., Izawa S., Hirakawa Y., Shimokata H., Akihisa I. (2011). Impact of caregiver burden on adverse health outcomes in community-dwelling dependent older care recipients. The American Journal of Geriatric Psychiatry: Official Journal of the American Association for Geriatric Psychiatry, 19, 382–391. 10.1097/JGP.0b013e3181e9b98d 20808120

[gnag163-B30] Laks J. , Goren A., Dueñas H., Novick D., Kahle-Wrobleski K. (2016). Caregiving for patients with Alzheimer’s disease or dementia and its association with psychiatric and clinical comorbidities and other health outcomes in Brazil. International Journal of Geriatric Psychiatry, 31, 176–185. 10.1002/gps.4309 26011093

[gnag163-B31] Lastuka A. , Breshock M. R., Taylor K. V., Dieleman J. L. (2025). The costs of dementia care by US state: Medical spending and the cost of unpaid caregiving. Journal of Alzheimer’s Disease: JAD, 105, 186–196. 10.1177/13872877251326231 PMC12231809 40111940

[gnag163-B32] Lawton M. P. , Brody E. M. (1969). Assessment of older people: Self-maintaining and instrumental activities of daily living. The Gerontologist, 9, 179–186. 10.1093/geront/9.3_part_1.179 5349366

[gnag163-B33] LeadingAge (n.d). *2026 policy platform*. https://leadingage.org/2026-policy-platform/

[gnag163-B34] Lee J. , Baik S., Becker T. D., Cheon J. H. (2022). Themes describing social isolation in family caregivers of people living with dementia: A scoping review. Dementia (London, England), 21, 701–721. 10.1177/14713012211056288 34872364

[gnag163-B35] Lee Y. , Choi W., Park M. S. (2022). Respite service use among dementia and nondementia caregivers: Findings from the National Caregiving in the U.S. 2015 Survey. Journal of Applied Gerontology: The Official Journal of the Southern Gerontological Society, 41, 1557–1567. 10.1177/07334648221075620 35303780

[gnag163-B36] Lee Y. , Xu L., Kim B. J., Chen L. (2020). Leisure activity, gender and depressive symptoms among dementia caregivers: Findings from REACH II. Aging & Mental Health, 24, 1886–1893. 10.1080/13607863.2019.1660853 31497991

[gnag163-B37] Lin X. , Moxley J. H., Czaja S. J. (2023). Caring for dementia caregivers: Psychosocial factors related to engagement in self-care activities. Behavioral Sciences (Basel, Switzerland), 13, 851. 10.3390/bs13100851 PMC10604240 37887501

[gnag163-B38] Liu Y. , Leggett A. N., Kim K., Polenick C. A., McCurry S. M., Zarit S. H. (2022). Daily sleep, well-being, and adult day services use among dementia care dyads. Aging & Mental Health, 26, 2472–2480. 10.1080/13607863.2021.1998354 PMC9109303 34761966

[gnag163-B39] Lund D. A. , Utz R., Caserta M. S., Wright S. D. (2009). Examining what caregivers do during respite time to make respite more effective. Journal of Applied Gerontology, 28, 109–131. 10.1177/0733464808323448

[gnag163-B40] Martindale-Adams J. , Nichols L. O., Zuber J., Burns R., Graney M. J. (2016). Dementia caregivers’ use of services for themselves. The Gerontologist, 56, 1053–1061. 10.1093/geront/gnv121 PMC5181390 26350152

[gnag163-B41] Mausbach B. T. , Roepke S. K., Depp C. A., Moore R., Patterson T. L., Grant I. (2011). Integration of the pleasant events and activity restriction models: Development and validation of a PEAR model. Behavior Therapy, 42, 78–88. 10.1016/j.beth.2009.11.006 PMC3163438 21292054

[gnag163-B42] Mensie L. C. , Steffen A. M. (2010). Depressive symptoms and use of home-based respite time in family caregivers. Home Health Care Services Quarterly, 29, 120–137. 10.1080/01621424.2010.511514 20845174

[gnag163-B43] Mossello E. , Caleri V., Razzi E., Di Bari M., Cantini C., Tonon E., Lopilato E., Marini M., Simoni D., Cavallini M. C., Marchionni N., Biagini C. A., Masotti G. (2008). Day care for older dementia patients: Favorable effects on behavioral and psychological symptoms and caregiver stress. International Journal of Geriatric Psychiatry, 23, 1066–1072. 10.1002/gps.2034 18481318

[gnag163-B44] Mushtaq D. M. R. , Alam I., Ali Z. A. (2025). How dementia stages influence the impact of stressors and caregiving appraisals on caregiver well-being. Social Science Review Archives, 3, 582–594. 10.70670/sra.v3i1.337

[gnag163-B45] NADSA (n.d). *National Adult Day Services Association*. https://www.nadsa.org/

[gnag163-B46] Nandi A. , Counts N., Bröker J., Malik S., Chen S., Han R., Klusty J., Seligman B., Tortorice D., Vigo D., Bloom D. E. (2024). Cost of care for Alzheimer’s disease and related dementias in the United States: 2016 to 2060. NPJ Aging, 10, 13. 10.1038/s41514-024-00136-6 PMC10853249 38331952

[gnag163-B47] Nasreen H. E. , Tyrrell M., Vikström S., Craftman Å., Syed Ahmad S. A. B., Zin N. M., Aziz K. H. A., Mohd Tohit N. B., Md Aris M. A., Kabir Z. N. (2024). Caregiver burden, mental health, quality of life and self-efficacy of family caregivers of persons with dementia in Malaysia. BMC Geriatrics, 24, 656. 10.1186/s12877-024-05221-9 PMC11301828 39103767

[gnag163-B48] Neville C. , Beattie E., Fielding E., MacAndrew M. (2015). Literature review: Use of respite by carers of people with dementia. Health & Social Care in the Community, 23, 51–53. 10.1111/hsc.12095 25602093

[gnag163-B49] Ng Y. T. , Freedman V., Kratz A., Birditt K. (2025). Beyond caregiving: Daily pleasant activities among caregivers to older adults with and without dementia. The Journals of Gerontology, Series B: Psychological Sciences and Social Sciences, 80, gbae198. 10.1093/geronb/gbae198 PMC13008407 39673747

[gnag163-B50] O’Shea E. , Timmons S., Fox S., Irving K. (2019). Respite in dementia: An evolutionary concept analysis. Dementia, 18, 1446–1465. 10.1177/1471301217715325 28659025

[gnag163-B51] Ogunjesa B. , Gao X., Raj M. (2024). Factors influencing caregivers’ use of respite care services. Journal of Applied Gerontology, 43, 1100–1110. 10.1177/07334648241229574 38298084

[gnag163-B52] Parker L. J. , Gaugler J. E., Samus Q., Gitlin L. N. (2019). Adult day service use decreases likelihood of a missed physician’s appointment among dementia caregivers. Journal of the American Geriatrics Society, 67, 1467–1471. 10.1111/jgs.15995 PMC6612582 31219175

[gnag163-B53] Parker L. J. , Gitlin L. N. (2021). Does adult day service use improve well-being of Black caregivers of people living with dementia? Innovation in Aging, 5, igab037. 10.1093/geroni/igab037 PMC8570428 34754949

[gnag163-B54] Pearlin L. I. , Mullan J. T., Semple S. J., Skaff M. M. (1990). Caregiving and the stress process. The Gerontologist, 30, 583–594. 10.1093/geront/30.5.583 2276631

[gnag163-B55] Radloff L. S. (1977). The CES-D scale: A self-report depression scale for research in the general population. Applied Psychological Measurement, 1, 385–401. 10.1177/014662167700100306

[gnag163-B56] Riffin C. , Van Ness P. H., Wolff J. L., Fried T. (2019). Multifactorial examination of caregiver burden in a national sample of family and unpaid caregivers. Journal of the American Geriatrics Society, 67, 277–283. 10.1111/jgs.15664 PMC6367031 30452088

[gnag163-B57] Robertson K. , Thyne M., Thomson R., Watkins L. (2025). The impact of social isolation and loneliness on the well-being of carers of a person with dementia in Aotearoa New Zealand. Dementia (London, England), 24, 235–248. 10.1177/14713012241279683 PMC11780974 39214523

[gnag163-B58] Rubin D. B. (2009). Multiple imputation for nonresponse in surveys. Wiley.

[gnag163-B59] Sadarangani T. , Fernandez Cajavilca M., Qi X., Zagorski W. (2024). Adult day services: A potential antidote to social isolation and loneliness in marginalized older adults. Frontiers in Public Health, 12, 1427425. 10.3389/fpubh.2024.1427425 PMC11412866 39310908

[gnag163-B60] Sadarangani T. R. , Gaugler J. E., Dabelko-Schoeny H., Marx K. A. (2022). Adult day services, health equity for older adults with complex needs, and the COVID-19 pandemic. American Journal of Public Health, 112, 1421–1428. 10.2105/AJPH.2022.306968 PMC9480461 36103694

[gnag163-B61] Scher C. J. , Anderson K., Zagorski W., Siamdoust S., Li Y., Sadarangani T. (2025). “We feel like a family here”: Person-centered outcomes of adult day services use. BMC Health Services Research, 25, 1188. 10.1186/s12913-025-13436-8 PMC12406401 40903739

[gnag163-B62] Schüz B. , Czerniawski A., Davie N., Miller L., Quinn M. G., King C., Carr A., Elliott K.-E. J., Robinson A., Scott J. L. (2015). Leisure time activities and mental health in informal dementia caregivers. Applied Psychology. Health and Well-Being, 7, 230–248. 10.1111/aphw.12046 26097155

[gnag163-B63] Sheehan O. C. , Haley W. E., Howard V. J., Huang J., Rhodes J. D., Roth D. L. (2021). Stress, burden, and well-being in dementia and nondementia caregivers. The Gerontologist, 61, 670–679. 10.1093/geront/gnaa108 PMC8276607 32816014

[gnag163-B64] Solimando L. , Fasulo M., Cavallero S., Veronese N., Smith L., Vernuccio L., Bolzetta F., Dominguez L. J., Barbagallo M. (2022). Suicide risk in caregivers of people with dementia: A systematic review and meta-analysis. Aging Clinical and Experimental Research, 34, 2255–2260. 10.1007/s40520-022-02160-6 PMC9637612 35696056

[gnag163-B65] Stevens A. B. , Coon D., Wisniewski S., Vance D., Arguelles S., Belle S., Mendelsohn A., Ory M., Haley W. (2004). Measurement of leisure time satisfaction in family caregivers. Aging & Mental Health, 8, 450–459. 10.1080/13607860410001709737 15511743

[gnag163-B66] Stirling C. M. , Dwan C. A., McKenzie A. R. (2014). Why carers use adult day respite: A mixed method case study. BMC Health Services Research, 14, 245. 10.1186/1472-6963-14-245 PMC4068069 24906239

[gnag163-B67] Tretteteig S. , Vatne S., Rokstad A. M. M. (2016). The influence of day care centers for people with dementia on family caregivers: An integrative review. Aging & Mental Health, 20, 450–462. 10.1080/13607863.2015.1023765 25815563

[gnag163-B68] Tretteteig S. , Vatne S., Rokstad A. M. M. (2017). The influence of day care centers designed for people with dementia on family caregivers: A qualitative study. BMC Geriatrics, 17, 5. 10.1186/s12877-016-0403-2 PMC5216603 28056843

[gnag163-B69] Turner S. G. , Mindlis I., Reid M. C., Pillemer K. A. (2024). Caregiving challenges from persistent pain among family caregivers to people with dementia. The Gerontologist, 65, gnae164. 10.1093/geront/gnae164 PMC11659591 39501427

[gnag163-B70] Utz R. L. (2022). Caregiver respite: An essential component of home and community-based long-term care. Journal of the American Medical Directors Association, 23, 320–321. 10.1016/j.jamda.2021.12.020 PMC9052870 34971592

[gnag163-B71] Utz R. L. , Caserta M., Iacob E., Sparks C., Stark L., Terrill A., Thompson A., Wong B. (2023). Maximizing the benefit of respite for dementia caregivers: Time for Living & Caring intervention protocol. OBM Integrative and Complementary Medicine, 08, 1–23. 10.21926/obm.icm.2304040 PMC10838170 38313766

[gnag163-B72] Vandepitte S. , Van Den Noortgate N., Putman K., Verhaeghe S., Verdonck C., Annemans L. (2016). Effectiveness of respite care in supporting informal caregivers of persons with dementia: A systematic review. International Journal of Geriatric Psychiatry, 31, 1277–1288. 10.1002/gps.4504 27245986

[gnag163-B73] Warren S. , Kerr J. R., Smith D., Schalm C. (2003). The impact of adult day programs on family caregivers of elderly relatives. Journal of Community Health Nursing, 20, 209–221. 10.1207/S15327655JCHN2004_02 14644688

[gnag163-B74] Wisniewski S. R. , Belle S. H., Marcus S. M., Burgio L. D., Coon D. W., Ory M. G., Burns R., Schulz R. (2003). The REACH project design and baseline characteristics. Psychology and Aging, 18, 375–384. 10.1037/0882-7974.18.3.375 PMC2577188 14518801

[gnag163-B75] Wylie M. J. , Kim K., Liu Y., Zarit S. H. (2021). Taking a break: Daily respite effects of adult day services. The Gerontologist, 61, 1231–1240. 10.1093/geront/gnaa178 PMC8679007 33416085

[gnag163-B76] Xu L. , Liu Y., He H., Fields N. L., Ivey D. L., Kan C. (2021). Caregiving intensity and caregiver burden among caregivers of people with dementia. Archives of Gerontology and Geriatrics, 94, 104334. 10.1016/j.archger.2020.104334 33516077

[gnag163-B77] Xu X. Y. , Kwan R. Y. C., Leung A. Y. M. (2020). Cardiovascular risk in family caregivers of people with dementia: A systematic review. Journal of International Medical Research, 48, 0300060519845472. 10.1177/0300060519845472 PMC7140198 31115265

[gnag163-B78] Young H. , Tonkikh O., Ogugu E., Famula J., Bell J. (2024). Supporting fortitude among family caregivers: The role of adult day services for persons with dementia. Innovation in Aging, 8, 239. 10.1093/geroni/igae098.0771

[gnag163-B79] Zarit S. H. , Bangerter L. R., Liu Y., Rovine M. J. (2017). Exploring the benefits of respite services to family caregivers: Methodological issues and current findings. Aging & Mental Health, 21, 224–231. 10.1080/13607863.2015.1128881 PMC5550302 26729467

[gnag163-B80] Zarit S. H. , Kim K., Femia E. E., Almeida D. M., Klein L. C. (2014). The effects of adult day services on family caregivers’ daily stress, affect, and health. The Gerontologist, 54, 570–579. 10.1093/geront/gnt045 PMC4155447 23690056

[gnag163-B81] Zarit S. H. , Stephens M. A., Townsend A., Greene R. (1998). Stress reduction for family caregivers: Effects of adult day care use. The Journals of Gerontology. Series B, Psychological Sciences and Social Sciences, 53, S267–S277. 10.1093/geronb/53b.5.s267 9750575

[gnag163-B82] Zarit S. H. , Whetzel C. A., Kim K., Femia E. E., Almeida D. M., Rovine M. J., Klein L. C. (2014). Daily stressors and adult day service use by family caregivers: Effects on depressive symptoms, positive mood and DHEA-S. The American Journal of Geriatric Psychiatry, 22, 1592–1602. 10.1016/j.jagp.2014.01.013 PMC4119567 24566240

[gnag163-B83] Zhu C. W. , Scarmeas N., Ornstein K., Albert M., Brandt J., Blacker D., Sano M., Stern Y. (2015). Health-care use and cost in dementia caregivers: Longitudinal results from the Predictors Caregiver Study. Alzheimer’s & Dementia: The Journal of the Alzheimer’s Association, 11, 444–454. 10.1016/j.jalz.2013.12.018 PMC4164583 24637299

