# Supplementary material for: Adult day services, leisure time satisfaction, and psychosocial well-being among dementia caregivers
Source: Gerontologist. 2026 Aug 6;66(10):gnag163. doi: 10.1093/geront/gnag163 (PMC13570679; doi:10.1093/geront/gnag163)
Supplement: Supplementary file 1 [file gnag163_supplementary_data.pdf]

Adult Day Services, Leisure Time Satisfaction, and Psychosocial Well-Being Among Dementia Caregivers

Clara J. Scher, Jerad Moxley, Catherine Riffin, Sara Czaja

Supplementary Table 1

Characteristics of the Studies Included in the Dataset

Study	Years of Data Collection	Geographic Location (U.S.)	Number of Participants	Recruitment Approach	Methods	Primary Funding Source
**REACH I**	~1995–2001	Multi-site, national (6 sites: Birmingham AL, Boston MA, Memphis TN, Miami FL, Palo Alto CA, Philadelphia PA)	~1,222 caregiver–care recipient dyads	Memory clinics, Alzheimer's Disease Centers, community agencies	Multi-arm randomized clinical trial of caregiver interventions	National Institute on Aging
**REACH II**	~2001–2004	Multi-site, national (Birmingham AL, Memphis TN, Miami FL, Palo Alto CA, Philadelphia PA)	~642 caregivers	Community and clinical recruitment targeting racial/ethnic diversity	Randomized controlled trial; multi-component psychosocial intervention vs usual care	National Institute on Aging
**Community REACH**	~2015–2016	South Florida (Miami-Dade County)	~146 caregivers	Community-based agencies	Implementation/translation of REACH II delivered by community providers	National Institute on Aging
**Video to Video Network**	~2010–2012	South Florida (Miami-Dade County)	~110 caregivers	Community recruitment, emphasis on Hispanic and African American caregivers	Videophone-delivered REACH II adaptation	National Institute on Aging
**Caring for the Caregiver Network**	~2017–2018	South Florida (Miami-Dade County)	~243 caregivers (baseline analytic sample)	Community outreach, caregiver organizations, advertisements	Technology-based caregiver study; baseline observational	National Institute of Nursing Research

					data used for secondary analyses	

Supplementary Table 2

Associations Between Leisure Time Satisfaction and Psychosocial Outcomes Across Separate Multivariable Mixed-Effects Models

	Estimate	Std. Error	t-value	df	P(>|t|)
Burden	-0.16	0.02	-8.83	7.31	0.000***
Depressive Symptoms	-0.02	0.01	-1.65	62.58	0.105
Social Support	0.21	0.01	17.18	140.14	0.000***
Loneliness	-0.04	0.01	-3.73	164.89	0.000***
Positive Aspects of Caregiving	0.04	0.01	2.88	29.30	0.007**

Note. ***=p<.001, **p<.01. Each estimate represents the association between leisure time satisfaction and the indicated psychosocial outcome from a separate multivariable mixed-effects regression model with a random intercept. All models controlled for the full set of covariates (e.g., caregiver and care recipient characteristics) and were estimated using multiply imputed data. Estimates reflect pooled results across five imputed datasets.
